# The ties that bind: Ingroup ties are linked with diminished inflammatory immune responses and fewer mental health symptoms through less rumination

**DOI:** 10.1371/journal.pone.0195237

**Published:** 2018-04-23

**Authors:** Renate Ysseldyk, Robyn J. McQuaid, Opal A. McInnis, Hymie Anisman, Kimberly Matheson

**Affiliations:** 1 Department of Health Sciences, Carleton University, Ottawa, Ontario, Canada; 2 Department of Neuroscience, Carleton University, Ottawa, Ontario, Canada; 3 The Royal’s Institute of Mental Health Research, affiliated with the University of Ottawa, Ontario, Canada; Montana State University, UNITED STATES

## Abstract

The present research explored whether components of social identity, namely ingroup ties, affect, and centrality, were differentially linked to mental health and inflammatory immune responses, and whether rumination mediated those relations. Study 1 (*N* = 138) indicated that stronger ingroup ties were associated with fewer mental health (depressive and post-traumatic stress) symptoms; those relations were mediated by the tendency for individuals with strong ties to rely less on ruminative coping to deal with a stressful life event. Study 2 (*N* = 54) demonstrated that ingroup ties were negatively associated with depressive symptoms, dispositional rumination, as well as stress-linked inflammatory elements at the physiological level. Consistent associations for centrality and ingroup affect were absent, suggesting that ingroup ties may have unique health benefits.

## Introduction

“*The ties that bind us to life are tougher than you imagine*, *or than any one can who has not felt how roughly they may be pulled without breaking*.”~ Anne Brontë

Trauma. Illness. Isolation. These are experiences that can tear us apart—both from within and from others with whom we share our lives. How does one cope with such stressors? What are the implications for health and well-being? An emerging research agenda based on the social identity perspective [1] [2] posits that a key factor in coping well with life’s trials and maintaining a positive state of health and well-being is rooted in one’s group memberships and social connections. These findings collectively suggest that a strong sense of group belonging—of social identification—is vital to maintaining good mental and physical health, especially in times of stress [3] [4] [5]. It appears that social identities serve both protective and curative functions associated with achieving good health [6]. What is often missing from this analysis, however, is consideration of how different dimensions of social identification may relate to various indices of physical and mental health, including the elicitation of coping strategies that may attenuate or exacerbate stress-related conditions.

Social identification refers to an individual’s “knowledge of his [or her] membership of a social group (or groups) together with the value and emotional significance attached to that membership” [7]. Although this definition taps into the cognitive (i.e., knowledge) and affective (i.e., emotional significance) dimensions of social identity, the extent to which such social groups offer a sense of connectedness with other group members—that is, “ingroup ties” [8] [9]—may be of particular importance in fostering health and well-being. In this regard, the elements of a social identity might operate differently, in part because of the role they play in facilitating access to key coping resources. Indeed, much of the coping research in the social identity tradition has, not surprisingly, focused on social support [3] [10] [11]; however, the propensity for social identification to also promote (or deter) other less obvious coping strategies to deal with life’s stressors has received comparatively less attention. For example, strong social ties might offset the tendency to engage in solitary *rumination* about one’s stressors, which is a coping strategy that has been found to be particularly damaging to health and well-being [12] [13] [14] [15] [16]. Thus, in the studies reported here, we explore how distinct components of social identification differentially relate to both mental health and related inflammatory immune responses, in part due to the coping strategies—namely rumination—with which they are linked.

### Ingroup ties and mental health

Social identification (in general) is linked to better mental health [17] [18] [19]. In addition to fostering positive psychological well-being more broadly [20], social group memberships can impact self-esteem [21] [22], guard against discrimination [23], and foster a sense of personal control [24] [25]. Social identification also appears to buffer against the negative effects of stress including chronic mental health disturbances, such as depression [26] and post-traumatic stress disorder (PTSD) [27] [28]. These benefits may depend, in part, on which group identity is salient. In this regard, religious group membership may have a broader range of benefits compared to some other groups due to its link not only to the social belonging associated with a religious identity, but as well, the belief systems that define worldviews [29] [30].

Most conceptualizations of social identification include both cognitive and affective components (following from Tajfel’s definition [7]). The cognitive component, often termed *identity centrality*, reflects the extent to which people consider their group identity to be an important part of their self-concept [8] [31] [32] whereas *ingroup affect* refers to the emotional evaluation that people feel towards their group [8] [9] [22] [31]. Another commonly considered component of social identification is the extent to which a common bond is shared with other group members. This sense of belonging and perception of common fate is captured in the notion of *ingroup ties* [8] [9]. Importantly, these components of identity may relate differently to stress-related health outcomes [33] and coping styles [34].

Few studies have attempted to disentangle the relations that exist among the components of social identification and their distinctive implications for stress-related outcomes. One notable example is a study conducted by Bombay, Matheson, and Anisman [33] in which the relations among specific aspects of ethnic identification (namely centrality, ingroup affect, and ingroup ties), discrimination, and depressive symptoms were assessed among First Nations adults in Canada. Both ingroup affect and ties buffered against perceived discrimination, and the former was also associated with decreased depressive symptoms. In contrast, identity centrality was associated with *increased* depressive symptomatology and intensified the relation between perceived discrimination and depressive symptoms. Similarly, psychological health was positively associated with ingroup ties, but was unrelated to either ingroup affect or identity centrality among a sample of Men’s Sheds users in Australia [35]. Given these incongruent relations with psychological health, it may be precarious to assume that composite scores of social identification will dependably reveal the relations that exist between identity and mental health; indeed, these identity components themselves may not always be linearly related [36]. It could be argued that in times of stress—when one needs a shoulder to cry on or an advocate to stand with—ingroup ties may be especially valuable in protecting health and well-being.

### Ingroup ties and inflammatory immune responses

Although there is considerable research demonstrating the importance of social identification for mental health, there is also evidence that physical health and resilience share this link [29] [37] [38] [39] [40]. Importantly, a significant association between the identity component of ingroup ties and physical health has also been demonstrated, whereas a link with either ingroup affect or identity centrality was absent [35]. However, although social identification may yield benefits in the face of acute physical health issues, of particular concern is whether or not it is linked to chronic health outcomes, which can have more detrimental implications for individuals long-term. Such a relation would suggest a more ubiquitous impact of social identification that might transcend specific situational contexts, and further serve as a social factor linking physical and mental health outcomes.

One pathway through which social identities may be related to physical (and mental) health conditions is chronic inflammation. In this regard, several studies have demonstrated that social factors such as isolation, loneliness, and fewer social network ties are associated with greater inflammation reflected by elevated C-reactive protein or levels of pro-inflammatory cytokines, such as tumor-necrosis factor-α (TNF-α) in adults with cancer, those at risk for developing cardiovascular disease, and in the general population [41] [42] [43] [44] [45] [46]. Of course, mental and physical health are inextricably linked, as so many of the same inflammatory processes acting on physical health also affect psychological symptoms. For example, although depression has been linked to several neurobiological processes, including various neurotransmitters, neurotrophins and hormones, activation of the inflammatory immune response is also linked to depressive symptoms [47] [48]. Indeed, elevated levels of TNF-α are associated with depressive symptoms [49] [50] [51]. Likewise, there is reason to believe that low levels of the *anti-*inflammatory cytokine interleukin-10 (IL-10) accompany high levels of depression [52]. To the extent that social identification (and ingroup ties in particular) acts as a stress buffer by enabling beneficial coping strategies, it is possible that it is related to circulating cytokine levels, which may reflect biological processes underlying depressive symptoms.

### The meditating role of rumination

Mental and physical health disturbances often exist alongside a variety of coping strategies used by individuals to deal with life’s stressors, namely problem-focused and emotion-focused coping [53] [54] [55]. Important variations exist in the relations between specific coping strategies and health and well-being, as well as how particular strategies are used depending on the resources proffered by the specific dimensions of identity. By example, the social support inherent in strong group ties is fairly intuitive, and may be especially useful in buffering the adverse effects of stressors and promoting health [35]. However, an exclusive focus on social support may ignore the propensity for social identification to promote other less obvious coping strategies to deal with life’s stressors.

Rumination, which typically entails self-reflection with a repetitive and passive focus on one’s negative emotions [56], may be especially vital to consider in the context of social identification. Rumination is typically regarded as a solitary and ineffective method for dealing with stressful experiences, and has been found to be among the strongest predictors of a number of stress-related psychological disturbances, including depression and PTSD [12] [13] [14] [15] [16] [57] [58]. However, a strong sense of belonging is associated with lower levels of both rumination and depressive symptoms [59]. In effect, although the social support inherent to ingroup ties may be the coping strategy typically assumed to help in reducing stress-related mental health disturbances, it may be through group members’ ability to draw the individual away from maladaptive forms of introspection and brooding—that is, rumination—through which this is accomplished.

### Overview of the present research

The present research investigated whether identity centrality, ingroup affect, and ingroup ties were differentially related to various indices of mental health and inflammatory immune markers, as well as rumination—which may be especially prone to attenuate or exacerbate stress-related health conditions. Study 1 assessed both rumination and social support among individuals who had previously experienced a traumatic life event, their identification with a valued social group (namely, religious group membership), and their mental health (symptoms of depression and PTSD). Study 2 expanded on this by examining whether there might be variations in the relations between the dimensions of identification (with a variety of social groups) and physiological markers, namely anti-and pro-inflammatory cytokines, as well as more specific components of rumination that may be differentially related to mental health. In so doing, the present research extends previous work in several important ways, most notably by (1) assessing links with specific *components* of social identification, (2) assessing both self-reported mental health symptoms and *physiological* stress reactions that may be differentially related to components of social identification, and (3) assessing the mediating role of *rumination*.

## Study 1

Study 1 examined associations between religious social identification (centrality, ingroup affect, ingroup ties) and mental health symptoms (depression and PTSD) among individuals who had experienced a previous traumatic life event. Although a strong sense of social identification may stem from any number of group memberships, in times of crises or insecurity, some group memberships might become especially salient. It has been argued that in situations that undermine an individual’s sense of safety and security, such as violent conflict or traumatic experiences, *religious* identity may become particularly important [60] [61] [62]. Perhaps identification with one’s religious group provides a sense of unwavering stability and “solid ground”, more so than might be gained from other social identities, as religion itself is perceived to offer answers in times of uncertainty and can provide order in the midst of chaos [60] [63]. Importantly, religious identification also often includes a network of close social ties (e.g., through attendance at worship services) [64] [65], and may be strongly related to a variety of coping methods [66].

Not surprisingly, the experience of trauma is associated with stress-related mental health symptoms [67]. However, social identities can buffer against the negative effects of traumatic stressors on symptomatology [26] [27]. Specifically, religious identity (and religious engagement) is associated with fewer mental health symptoms among survivors of trauma [68] [69]. This link may depend on a variety of factors, including the coping strategies on which individuals rely to deal with the stressor. Although social support is typically assumed to play a leading role in this regard, (less) rumination may also substantially contribute to linking social identities with better mental health. Thus, the potential mediating roles of both rumination and social support in the relations between religious social identity and mental health symptoms were assessed.

Accordingly, in Study 1, participants reported on identification with their religious group (including the three components of identity centrality, ingroup affect, and ingroup ties), coping strategies (namely social support and rumination) used to deal with a previous traumatic experience, and mental health symptoms (depressive and PTSD). It was hypothesized that the three dimensions of social identification would be differentially related to depressive and PTSD symptomatology. Specifically, it was expected that centrality would be unrelated, but that ingroup affect—and especially ingroup ties—would be negatively related to mental health symptoms. It was further expected that these relations would be mediated by individuals’ propensities to endorse social support seeking, and to *not* endorse rumination to cope with their traumatic experience.

### Methods

#### Participants and procedure

Undergraduate students were recruited to participate in a study on “coping with stressful life events”; *N =* 138; 99 women, 39 men; *M*_age =_ 21.18, *SD =* 6.02). Participants were provided with a study overview and, after giving written informed consent, completed questionnaires assessing religious group affiliation and identification, rumination, social support seeking, depressive and PTSD symptoms. They were subsequently debriefed and provided with contact numbers that included psychological services. Participants received course credit or $10 for their participation. Given the study’s focus on previous traumatic experiences, participants who indicated in a pre-measure (Traumatic Life Events Questionnaire, short-form) [70] administered to all first- and second-year psychology undergraduates that they had experienced such an event were over-sampled by inviting those who answered affirmatively to participate in the study. All study procedures were approved by Carleton University’s Research Ethics Board.

#### Measures

**Previous trauma.** The Traumatic Life Events Questionnaire (TLEQ) [70] assessed experiences of a range of traumatic events. These included: trauma resulting in shock (e.g., a car accident; *n* = 37), death of a loved one (*n* = 32), physical assault/abuse (*n* = 29), and trauma to another (e.g., witnessing abuse; *n* = 16). Only 32 individuals had not experienced some type of trauma and were instead asked to reflect on their most stressful life experience (e.g., a serious animal bite; being lost in the wilderness). The sum of the *n*s indicating trauma type >*N* = 138 as some individuals had experienced more than one type of traumatic event. Participants also indicated their age when their most traumatic/stressful life event occurred (or most recently occurred if the experience was ongoing or had occurred more than once); although the timeline for these experiences ranged from 0 to 17 years before participants’ completion of our questionnaires, on average, this event happened approximately four years ago (*M* = 3.78; *SD* = 3.56).

**Group identification.** Identification was measured using Cameron’s [8] 12-item tripartite scale, adapted to be in reference to participants’ religious identity. Items were rated on a scale ranging from 0 (“strongly disagree”) to 5 (“strongly agree”). Each subscale was assessed with 4 items, including identity *centrality* (e.g., “In general, being a member of my religious group is an important part of my self-image”; *M =* 1.98, *SD =* 1.28; α = .71), ingroup *affect* (e.g., “In general, I’m glad to be a member of my religious group”; *M =* 3.56, *SD =* 1.08; α = .71), and ingroup *ties* (e.g., “I feel strong ties to other members of my religious group”; *M =* 2.83, *SD =* 1.18; α = .71). Correlations among the subscales were moderate (Table 1).

**Table 1:** Inter-correlations among Study 1 variables.

	2	3	4	5	6	7
**Identity**						
1. Centrality	.60***	.65***	-.23**	.07	-.13	-.01
2. Affect	—	.64***	-.12	.03	-.29***	-.10
3. Ties		—	-.16^+^^a^	.07	-.26**	-.18*
**Coping**						
4. Rumination			—	.39***	.33***	.39**^b^
5. Social support				—	.05	.11
**Mental health**						
6. Depression					—	.50***
7. PTSD						—

^+^*p* = .07

*p < .05

**p < .01

***p < .001

^a^ The marginal negative correlation between ingroup ties and rumination becomes statistically significant (*p* = .036) using a one-tailed test, consistent with the hypothesized direction of the relation.

^b^ The potential also existed for shared process of negative repetitive thoughts between ruminative coping and the PTSD intrusiveness subscale; however, correlations with each of the subscales indicated that this was neither particularly the case for intrusiveness (*r* = .40, *p* < .001) nor the other PTSD subscales (avoidance, *r* = .33; hyperarousal, *r* = .39; ps < .001).

**Rumination and social support.** We employed the rumination and social support seeking subscales of the Survey of Coping Profile Endorsements (SCOPE) [54]. This scale includes 3 items that reliably assess rumination (i.e.,“Worried about your problems a lot”; “Thought about your problems a lot”; “Gone over your problems in your mind over and over again”), and 4 items assessing social support seeking (i.e., “Talked with friends or relatives about your problem”; “Sought the advice of others to resolve your problem”; “Asked others for help”; “Sought reassurance and moral support from others”). Respondents rated each item using a 5-point scale ranging from 0 (“never”) to 4 (“almost always”) in terms of whether they used each strategy to deal with their most stressful and/or traumatic life event (rumination: *M =* 2.23, *SD =* 1.13; α = .90; social support: *M =* 2.03, *SD =* 1.08; α = .89). Although the SCOPE also includes several other coping subscales (self- and other-blame, emotional expression and containment, activity and passivity, denial, cognitive restructuring, problem-solving, humour, wishful thinking and religious coping), rumination and social support were of particular interest in Study 1, given their links with depressive and PTSD symptoms as well as ingroup ties [12] [13] [14] [15] [16] [57] [58] [59].

**Depressive symptoms**. The 21-item Beck Depression Inventory (BDI) [71] assessed depressive symptoms. Participants were asked to select from sets of response choices reflecting increasing degrees of problem severity (e.g., from 0 ("I do not feel sad") to 3 ("I am so sad or unhappy that I can't stand it"), varying in number from 4 to 6 response choices. Responses were summed to provide a single index of depressive symptomatology, such that higher scores reflected greater depressive symptoms (*M =* 8.59, *SD =* 8.43; α = .92).

**PTSD symptoms.** The 22-item Impact of Events Scale (IES-R) [72] assessed PTSD symptomatology. Using a 5-point scale ranging from 0 (not at all) to 4 (extremely), participants rated how distressing each symptom had been during the past 7 days as a result of their most severe prior trauma (e.g., natural disaster, death of a loved one, assault/abuse, etc.). Although the IES-R assesses three dimensions, including intrusiveness (e.g., “Pictures about it popped into my mind”), avoidance (e.g., “I tried to remove it from my memory”), and hyperarousal (e.g., “I was jumpy and easily startled”), the correlations among them were sufficiently high (*r*s ranged from .79 to .88, *p*s < .001) to warrant creating a single summed score of PTSD symptoms (*M =* 18.82, *SD =* 20.13; α = .96).

#### Statistical analyses

Preliminary analyses indicated that the assumptions of our planned analyses (e.g., normality, outliers, homoscedasticity) were met, with few exceptions (see details in S1 Appendix). Pearson correlations assessed the relations among the variables of interest, and we subsequently conducted mediation analyses to determine whether rumination and social support accounted for the relations between social identification (i.e., centrality, ingroup affect, ingroup ties) and mental health (i.e., symptoms of depression and PTSD). To assess these mediated models, we conducted regression-based bootstrapping analyses with 95% confidence intervals (CIs) using Hayes’ PROCESS macro [73] [74]; this analysis provides evidence of mediation if the significant direct effect between the predictor and outcome variable (*c* path) is reduced when the mediator(s) is included (*c*’ path) and the 95% CI does not include zero [75].

### Results and discussion

As seen in Table 1, of the identity subscales, only centrality and ingroup ties were associated with rumination. Both ingroup affect and ties were associated with fewer depressive symptoms, but ingroup ties was the only component of group identification to be associated with fewer PTSD symptoms. As predicted, rumination was significantly related to depressive and PTSD symptoms. Interestingly, however, social support was neither related to social identification nor mental health. Nonetheless, the potential mediating role of rumination and social support in the relations between group identification and mental health symptoms were evaluated for those models wherein the specific identity component (i.e., centrality, affect, or ties) and mental health outcome (i.e., depressive or PTSD symptoms) were significantly related.

#### The mediating role of rumination

When the mediating roles of rumination and social support were assessed in the relation between ingroup *affect* and depressive symptoms, the direct relation (*c* path) [74] [75], β = -.29, *p* < .001, remained significant (*c*’ path), β = -.25, *p =* .002. An indirect path between ingroup affect and depressive symptoms through rumination, β = .33, *p <* .001, but not social support, β = -.08, *p* = .382, was also significant. However, the 95% CI for the mediated paths through both rumination, *B =* -0.31, SE = .22, 95% CI = -0.85, 0.07, and social support included zero, *B =* -0.02, SE = .08, 95% CI = -0.27, 0.08. Thus, neither rumination nor social support appeared to play a mediating role in the tendency for individuals with higher levels of ingroup affect to report fewer depressive symptoms.

When the mediating roles of rumination and social support were assessed in the relation between ingroup *ties* and depressive symptoms, the direct relation, β = -.26, *p =* .002, remained significant, β = -.20, *p =* .013, and once again rumination, β = .33, *p <* .001, but not social support, β = -.07, *p* = .443, appeared to provide an indirect path between this aspect of identity and depressive symptoms. Moreover, the 95% CI for rumination’s mediated path did not include zero, *B =* -0.37, SE = .23, 95% CI = -0.94, -0.01, whereas the mediated path for social support did, *B =* -0.03, SE = .07, 95% CI = -0.30, 0.05. Thus, in contrast to ingroup affect, rumination partially mediated the relation between ingroup ties and depressive symptoms.

When the mediating roles of rumination and social support were assessed in the relation between ingroup ties and PTSD symptoms, the direct relation, β = -.18, *p =* .038, was reduced to non-significance, β = -.12, *p =* .149, and rumination, β = .39, *p <* .001, but not social support, β = -.04, *p* = .682, appeared to account for that decrease. In this regard, the 95% CI for rumination’s mediated path did not include zero, *B =* -1.00, SE = .63, 95% CI = -2.55, -0.04, whereas the mediated path for social support did, *B =* -0.04, SE = .20, 95% CI = -0.82, 0.23. Although alternative models were examined (e.g., the mediating role of rumination in depressive symptoms predicting ingroup ties), none were significant.

In sum, although religious identity centrality was associated with a lower reliance on coping through rumination, it was not associated with depressive or PTSD symptoms. Similarly, although ingroup affect was related to less depressive symptomatology, it was not associated with rumination or PTSD symptoms. In contrast, stronger ingroup ties were associated with a reduced propensity to cope using rumination, as well as fewer depressive and PTSD symptoms. Moreover, the relations between ingroup ties and mental health were in part accounted for by the reluctance to engage in ruminative coping strategies, but the same patterns were not evident with regard to seeking social support in an effort to cope. In effect, the findings of the present study suggest that the presence of strong (religious) ingroup ties may yield the greatest benefits for adaptive coping—namely rumination—and, in turn, mental health.

## Study 2

Study 1 provided support that both ingroup ties and rumination may be especially valuable to consider in the link between religious social identification and mental health outcomes. However, such outcomes might differ among non-religious individuals, or with respect to different types of social groups more generally. A better understanding of the underlying physiological processes, and in particular inflammatory processes, that might be implicated would also provide further insights into the mechanisms underlying such relations [46]. It is possible that effective coping methods are associated with diminished levels of pro-inflammatory cytokines or high levels of anti-inflammatory factors, which have been linked to social ties in previous research [76] [77] [78]. In the same vein, both inflammatory processes and feelings of social disconnection are known to contribute to depressive symptoms [47] [48] [49], as well as to the incidence of numerous chronic physical health problems [76] [77] [78]. Thus, in Study 2 it was of interest to assess whether circulating cytokines (namely pro-inflammatory TNF-α and anti-inflammatory IL-10) and mental health (i.e., depressive) symptoms would be linked to social identity (and, in particular, ingroup ties), which can act as a stress buffer. In addition, although identification with one’s religious group was assessed in Study 1, the extent to which our findings apply to other group memberships was examined in Study 2.

Given the results of Study 1, and the preponderance of other research demonstrating the importance of rumination [12] [13] [14] [15] [58], we expanded our measurement of this construct. In Study 2, we examined rumination as a multidimensional dispositional coping style [56]. Indeed, although one might engage in ruminative coping in response to an acute event, it can also be thought of as a relatively stable coping style, rendering those who use it on a repeated basis more vulnerable to chronic mental and physical health challenges [79]. This said, several variants of rumination exist, and some may be more detrimental to health than others. In this regard, rumination has been conceptualized to encompass several dimensions, including ruminative depression (cognitive and emotional components related to depression), brooding (moody pondering and criticism of self and others), and reflection (contemplating the basis for one’s feelings, which might even be beneficial) [56] [80]. Thus, following from Study 1, it was expected in Study 2 that ingroup ties with a variety of social groups (religious and otherwise) would be most strongly associated with depressive symptoms and circulating cytokines, and that these relations would be mediated by dimensions of a ruminative coping style (and especially depressive or brooding rumination).

### Methods

#### Participants and procedure

Sixty-three undergraduate women participated in the study; however, 9 participants experiencing cold symptoms were removed from analyses due to potential effects on circulating cytokine levels (*N =* 54; *M*_age =_ 19.52, *SD =* 2.20). All study procedures were approved by Carleton University’s Research Ethics Board and Biohazard Committee.

**Laboratory session.** Laboratory sessions were held between 1300 and 1730 h, and it was required that women did not eat, drink (with the exception of water) or smoke for at least one hour prior to the session. Participants provided written informed consent and then completed a series of questionnaires assessing demographic information, social identity, rumination, and depressive symptomatology. This permitted a 30-min habituation period to the laboratory environment. Following completion of these questionnaires, a registered nurse drew a blood sample for later cytokine detection.

**Blood collection.** A blood sample was collected into a chilled EDTA coated vacutainer tube. Following collection, the sample was centrifuged for 15 min at 4°C and 2100 g, plasma was immediately aliquoted into Eppendorf tubes and frozen at −80°C.

#### Measures

**Group identification.** As in Study 1, group identification was measured using Cameron’s [8] 12-item scale, which included identity *centrality* (*M =* 3.21, *SD =* 1.23; α = .78), ingroup *affect* (*M =* 4.49, *SD =* 0.69; α = .81), and ingroup *ties* (*M =* 4.04, *SD =* 0.86; α = .72). In this case, however, participants reported their levels of group identification with the group that they perceived to be most important to their self-concept. Responses primarily referred to identifying as a student or member of a student sorority group (*n =* 18), as a woman (*n =* 13), with one’s religion and/or ethnicity (*n =* 9), as an athlete (*n =* 5), or some combination of these (e.g., female athlete; *n =* 6). Correlations among the three identity subscales were moderate (Table 2).

**Table 2:** Inter-correlations among Study 2 variables.

	2	3	4	5	6	7	8	9
**Identity**								
1. Centrality	.23^+^	.36**	.29*	.00	.07	-.09	.34*	-.06
2. Affect	—	.49^***^	.15	-.10	-.05	-.07	.08	-.34*
3. Ties		—	.09	-.37**	-.30*	-.30*	.37**	-.42**
**Rumination**								
4. Reflective			—	.41**	.51***	.19	.10	.01
5. Depressive				—	.79***	.72***	-.08	.24^+^
6. Brooding					—	.51***	-.07	.13
**Mental health**								
7. Depression						—	-.19	.22
**Circulating cytokines**								
8. IL-10							—	-.20
9. TNF-α								—

^+^*p* < .10

*p < .05

**p < .01

***p < .001

**Rumination.** The Ruminative Responses Scale [56] is a 22-item measure that assesses each of the ruminative reflection (5 items; *M =* 1.91, *SD =* 0.65; α = .65), brooding (5 items; *M =* 2.12, *SD =* 0.77; α = .82) and depression-related (12 items; *M =* 2.00, *SD =* 0.69; α = .91) components of rumination. Participants rated each item from 1 (“almost never”) to 4 (“almost always) in terms of what they generally do when they feel down, sad, or depressed. Not surprisingly, the rumination subscales were significantly and positively inter-correlated (*r*s ranged from .41 to .79).

**Depressive symptoms.** The 21-item BDI [71] assessed depressive symptoms. Responses were again summed to provide a single index of depressive symptomatology (*M =* 6.61, *SD =* 5.78; α = .86).

**Plasma cytokine assays**. Circulating levels of TNF-α and IL-10 were determined in duplicate by ELISA using human TNF-α and IL-10 high sensitivity kits obtained from R & D Systems (Minneapolis, MN). The assays were performed according to the manufacturer’s protocols. The intra- and inter-assay variability was less than 10% for both cytokines. Furthermore, the sensitivity was 0.19pg/mL and 0.17pg/mL for the TNF-α and IL-10 assays, respectively.

#### Statistical analyses

As in Study 1, preliminary analyses indicated that the assumptions of our planned analyses (e.g., normality, outliers, homoscedasticity) were met, with few exceptions (see details in S1 Appendix). The same analytic strategy was used in Study 2 (e.g., Pearson correlations, PROCESS mediation analyses) [73] [74] [75] to determine whether the three rumination subtypes differentially accounted for relations between social identification (i.e., centrality, ingroup affect, ingroup ties) and mental health (i.e., symptoms of depression) or circulating cytokines (TNF-α and IL-10).

### Results and discussion

As seen in Table 2, identity centrality was positively associated with ruminative reflection, whereas ingroup ties were negatively associated with ruminative depression, brooding, and depressive symptoms. Ingroup affect was not associated with any of the rumination subscales or with depressive symptoms.

Also shown in Table 2, unexpectedly, levels of IL-10 and TNF-α were not significantly related to rumination or depressive symptoms; however, IL-10 was positively related to ingroup ties and centrality. As well, TNF-α levels were negatively related to ingroup ties and to identity affect. Thus, only ingroup ties were associated both with greater circulating levels of the anti-inflammatory cytokine IL-10 and lower levels of the pro-inflammatory cytokine TNF-α.

#### The mediating role of rumination

When the potential mediating roles of the rumination subscales were assessed in the relation between ingroup ties and depressive symptoms, the direct relation (*c* path) [74] [75], β = -.30, *p =* .032, was reduced to non-significance (*c*’ path), β = -.02, *p =* .889; only ruminative depression appeared to contribute to that decrease, β = .84, *p* < .001, and the 95% CI for ruminative depression’s mediated path did not include zero, *B =* -2.06, SE = .89, 95% CI = -4.26, -0.56. Thus, the tendency for individuals with strong ingroup ties to report lower depressive symptoms was largely due to their disinclination to engage in depressive ruminative thinking. Although it could be argued that depressive rumination is both a “concomitant and predictor” (p. 671) [57]of depressive symptoms, it is equally arguable that ruminative brooding (i.e., comprising moody pondering and self-criticism) could play such a dual role. Indeed, in the present study, although ruminative brooding was not a significant mediator, it was nonetheless positively related to depressive symptoms and negatively related to ingroup ties.

When the mediating roles of the rumination subscales were assessed in the relations between ingroup ties and IL-10, and TNF-α, none reached significance, most likely due to the lack of relations among rumination and these inflammatory factors (i.e., the *b* paths) [74] [75], despite the strong associations these inflammatory markers shared with ingroup ties (i.e., the *a* paths) [74] [75]. Likewise, when the mediating roles of the rumination subscales were assessed in the relations between identity centrality or ingroup affect and inflammation, none were significant.

To summarize, as in Study 1, ingroup ties appeared to have consistent negative associations with mental health symptoms as well as circulating cytokines that were not shared by the other components of group identification. Given these findings, it is tempting to speculate that having poor ingroup ties might promote a pro-inflammatory bias, which has been previously linked to depression [48], as well as a variety of physical health conditions [76] [77] [78]. However, given the correlational nature of these data, it may be premature to draw such conclusions. Nonetheless, the results of the present study provide some preliminary evidence that ingroup ties may be linked to immunological factors, which complements the links found in the previous study between ingroup ties and self-reported mental health.

Although rumination was not associated with inflammatory factors, this coping style was once again important in mediating the relation between ingroup ties and depressive symptoms. In this case, however, given the multi-dimensional measure of rumination we employed, we were able to further disentangle the pathway through which this relation might exist. In effect, although strong ties with other group members were directly associated with fewer depressive symptoms, this appeared to be accomplished primarily through a reduction in ruminative depression (i.e., dwelling on the bases and symptoms of one’s depression)—rather than through ruminative brooding (i.e., moody pondering and self-criticism) or even the more constructive style of ruminative reflection (i.e., akin to problem-solving). Thus, the findings of Study 2 are in keeping with the results of Study 1 highlighting the important role of rumination in linking social ties with mental health.

## General discussion

Together, the findings of the two studies reported here suggest that ingroup ties may be an essential element in the link between social identification and health [6].[20]. We were particularly interested in the differential relations that specific components of social identification—namely centrality, ingroup affect, and ingroup ties—might have with various mental health outcomes, as well as the role of rumination in mediating those relations. As expected, strong ingroup ties were associated with fewer self-reported symptoms of both depression and PTSD. This was found across both studies (and with similar magnitudes of effect size), irrespective of stressor (traumatic or not), and with a variety of group memberships (e.g., religion, gender, sporting group, etc.). Similarly, ingroup ties were especially related to reduced inflammatory factors that could potentially underlie chronic mental and physical health conditions. Finally, rumination (but not social support) mediated the relations between ingroup ties and mental health symptoms in both studies. Given that these associations were not consistently evident with regard to identity centrality or ingroup affect, the present research makes an important contribution toward disentangling how distinct components of social identification may relate to health and well-being.

### Implications for social identities and health

Just as there were inconsistent outcomes associated with centrality and ingroup affect reported here, such disparities exist across the literature. These disparities have been especially evident with regard to the centrality dimension of social identification, which has been either negatively associated [33] or unrelated to health outcomes [35]. Indeed, centrality was largely unrelated to mental health outcomes in both studies here, with the exception of its positive link with the anti-inflammatory cytokine IL-10 in Study 2, suggesting that identity centrality might be linked to health conditions associated with inflammation. Although centrality was associated with a reduced inclination to ruminate following trauma in Study 1, this association was not carried through in Study 2. Given that the social identity assessed in Study 1 was specific to religious group membership, it may be that having a central *religious* identity—encompassing both belief-based and social elements—is especially helpful in coping with life’s stressors [62], whereas these benefits were diluted in the subsequent study when multiple group memberships were assessed. To be sure, when the construct of rumination itself was examined in more depth, centrality based on a variety of group memberships was *positively* associated with ruminative reflection. While it has been suggested that this dimension of rumination may be a more constructive form of this coping strategy [56], such evidence is tenuous—ruminative reflection has also been found to be positively associated with depressive symptoms [16] and was unrelated to mental health in the present research.

The relations between ingroup affect and depressive symptoms were more promising, albeit again inconsistent. For example, although ingroup affect was associated with fewer depressive symptoms in Study 1 following recall of a traumatic incident, relations with mental health and rumination were lacking in Study 2 (wherein no specific stressor was recalled). It may be that ingroup affect (which entails feelings of group pride) yields the greatest mental health benefits in emotionally-charged situations, rather than in response to more common daily occurrences. However, in Study 2, ingroup affect was associated with lower levels of the pro-inflammatory cytokine TNF-α, corroborating earlier research suggesting that feeling good about one’s group may have some benefits toward reducing inflammation (or alternatively, that having low levels of TNF-α might make one feel good about one’s group membership) [49]. Nevertheless, the lack of consistent associations between ingroup affect and mental health outcomes in the present research suggest that this component of social identification may not be the primary driving force behind the social cure [6].

In contrast, ingroup ties were dependably associated with fewer mental health symptoms, a lower reliance on rumination as a strategy for coping, as well as beneficial links with (pro- and anti-) inflammatory cytokines. Of particular note was the link between strong ingroup ties and fewer symptoms of PTSD. Although much research has examined the link between social identification and depressive symptoms [26] [81] fewer studies have considered PTSD symptomatology (cf. [27] [28]). In the present research, ingroup ties—and in particular ties with a religious group—was the only dimension of social identification that was linked with PTSD symptoms, wherein participants responded to a previous traumatic event (Study 1). Thus, it may be that in times of severe stress, health benefits are maintained through strong social ties, which provide a sense of belonging and the perception of a common bond with other group members, especially when those group members also share a guiding worldview. Moreover, ingroup ties was the only element of social identification to be associated with fewer depressive symptoms in Study 2. Thus, even with regard to this more well-established link between social identification and mental health, the results of the present research suggest that ingroup ties may be a fundamental component in lower depressive symptomatology.

Alongside fewer mental health symptoms, ingroup ties were also uniquely associated with *both* higher levels of anti-inflammatory IL-10 and lower levels of pro-inflammatory TNF-α (Study 2). These findings are in line with previous research in which strong social ties have been linked to better health outcomes, including cancer [82] [83], arthritis [84], and other inflammatory conditions [85]. Interestingly, however, in contrast to earlier research [47] [48] [49] [50] [51] [52], depressive symptoms were unrelated to the inflammatory markers assessed, with the exception of a modest relation between depressive symptoms and TNF-α. And yet, ingroup ties were associated both with fewer depressive symptoms and inflammatory cytokines. Although an examination of the mechanisms through which these factors may be intertwined was beyond the scope of the present research, our findings highlight the potential role of the ingroup ties dimension of social identification in managing both depressive symptoms and inflammation.

#### Implications for coping factors linking social identities and health

As noted earlier, several factors have been demonstrated to mediate the relation between social identification and health, including a sense of personal control [24], self-stereotyping [86], and especially social support [3] [10] [11]. A goal of the present research was to explore the role of rumination (in addition to social support), in that it might be especially sensitive to the nature of individuals’ connections to social groups. In this regard, rumination, both as a coping strategy in response to a specific stressor (Study 1) and as a dispositional coping style (Study 2), served as a pathway linking ingroup ties and PTSD symptomatology in Study 1, and with depressive symptoms in both studies. Given the emotion-focused nature of rumination, it might not be surprising that the rejection of this coping strategy accounted for the fewer mental health symptoms reported by individuals with strong ingroup bonds. Indeed, the emotional support often offered by one’s ingroup members, as well as the more active social participation inherent in strong ties, may serve as both a comfort and a distraction from brooding about past events. However, somewhat surprisingly, this was not the case for social support seeking in itself, suggesting that the benefits of social identification (and ingroup ties, in particular) are not necessarily about actively seeking advice or moral support from others, but may also stem from a more positive collective frame of mind. Taken together, these findings further support the idea that ingroup ties may play a unique role in promoting adaptive coping (or, in this case, discouraging maladaptive coping) and, in turn, fostering greater mental health across a variety of contexts.

### Caveats and conclusions

Some limitations of the present research should be noted, including that the sample size in Study 2 was relatively small, in part due to logistical constraints in data collection (i.e., the presence of a nurse required for blood samples). In addition, the sample comprised only women, given that women typically report higher levels of both rumination and depression [87] and to reduce potential variability based on physiological differences across the sexes [88]. However, it is worth noting that our effect sizes were comparable to those in other published research assessing inflammatory factors (i.e., cytokines) in human samples [89] [90]. Nonetheless, these findings can be considered provisional to further research assessing potential gender differences with larger samples.

Despite the significant relations between ingroup ties and both TNF-α and IL-10, no mediated relations were found. Based on the strength of association between ingroup ties and ruminative depression, as well as between ruminative depression and depressive symptoms (controlling for ingroup ties), our sample size was adequate to detect a mediated effect [91]. However, given the lack of significant relations between rumination and inflammatory cytokines, this may not have been the case when the latter variables comprised the outcomes in our meditational analyses. Of course, it may also be that other factors (not assessed in the present research; e.g., sleep disturbances [92], socio-economic adversity [93]) are more important in mediating the relation between social identification and inflammatory factors. Additionally, our data are primarily correlational, and comprised self-report surveys administered to undergraduate student samples. Thus, causal conclusions and generalizations to broader populations should be made cautiously (e.g., more depressed participants may be less inclined to seek out or to maintain ingroup ties, rather than those ties necessarily reducing depressive symptoms [49]). Despite these caveats, the convergence of patterns across studies provides some confidence about their robustness.

Indeed, the present research also has several strengths, including the use of different (situational vs. dispositional) coping measures, as well as physiological indicators—namely, (anti-)inflammatory cytokines. Across both studies, the consistent links between ingroup ties and mental health became evident, as was the mediating role of rumination in those relations. Overall, the present research points to the importance of considering distinct components of social identification, and of examining the role of specific coping strategies—rumination, in particular—in illuminating how the benefits of social identification might be realized. As Anne Brontë has noted, of particular import is the task of preserving the *ties that bind* group members together, which may be uniquely linked to adaptive coping strategies as well as fewer stress-related mental health and inflammatory conditions.

## Supporting information

S1 Appendix Preliminary analyses. (DOCX)

S1 Data Study 1 data. (SAV)

S2 Data Study 2 data. (SAV)
