# Supplementary material for: The ties that bind: Ingroup ties are linked with diminished inflammatory immune responses and fewer mental health symptoms through less rumination
Source: PLoS One. 2018 Apr 23;13(4):e0195237. doi: 10.1371/journal.pone.0195237 (PMC5912761; doi:10.1371/journal.pone.0195237)
Supplement: S1 Appendix — (DOCX) [file pone.0195237.s001.docx]

**S1 Appendix: Preliminary Analyses**

Preliminary analyses for Study 1 indicated that the assumptions of our planned analyses were met, with the exceptions of positively skewed distributions (i.e., skewness and kurtosis values beyond +/- 1; George & Mallery, 2010) for depression (skewness=1.335; kurtosis=1.248) and PTSD (skewness=1.184; kurtosis=0.715), such that more participants reported relatively few symptoms of depression or PTSD, as has been previously and commonly reported among measures of negative affect (Merz et al., 2013). Log transformation of the depression (skewness= -0.267; kurtosis=-0.523) and PTSD (skewness=-0.694; kurtosis=-0.195) variables corrected the skewness of the distributions; however, given that some cases were equal to zero on these variables, this transformation resulted in missing data for several participants. There were also 2 outliers (>3 *z*-scores) on depressive symptoms and 1 outlier on PTSD symptoms. Removal of the outliers and analyses using the log-transformed data revealed the same patterns of results as with the untransformed data (i.e., the magnitude and significance of correlations and mediated relations remained comparable), with the exception of a significant negative correlation that emerged between religious identity centrality and the log transformation of depressive symptoms (*r*=-.22, *p*=.018).

Preliminary analyses for Study 2 also indicated that the distribution for depressive symptoms was positively skewed (skewness=1.117; kurtosis=1.657), as was the distribution (slightly) for ruminative depression (skewness=1.019; kurtosis=0.932). The distribution for ingroup affect was negatively skewed (i.e., most people felt fairly good about their group membership; skewness=-2.016; kurtosis=4.740), suggesting that this commonly reported phenomenon regarding negative affect (Merz et al., 2013) might extend to group-level affect. There was also one outlier (*z*-score=-3.20) on ingroup affect. Log transformation of the depressive symptoms (skewness= -0.575; kurtosis=-0.856) and ruminative depression (skewness=0.094; kurtosis=-0.604) variables corrected the skewness of these distributions; however, even with removal of the outlier, the skewness of the ingroup affect distribution remained (skewness= -2.457; kurtosis=-8.322) such that most people felt good about their group memberships. Removal of the outlier and analyses using the log-transformed data revealed the same patterns of results as with the untransformed data (i.e., the magnitude and significance of correlations and mediated relations remained comparable). Thus, for ease of interpretation, results using untransformed data for both studies are presented here.
